# High-throughput transcriptomics

**DOI:** 10.1038/s41598-022-23985-1

**Published:** 2022-11-29

**Authors:** Nunzio D’Agostino, Wenli Li, Dapeng Wang

**Affiliations:** 1 Department of Agricultural Sciences, University of Naples Federico II, Portici, NA Italy; 2 Dairy Forage Research Center, USDA-ARS, 1925 Linden Drive, Madison, WI 53706 USA; 3 National Heart and Lung Institute, Imperial College London, London, SW3 6LY UK

**Keywords:** Computational biology and bioinformatics, Molecular biology

## Abstract

High-throughput transcriptomics has revolutionised the field of transcriptome research by offering a cost-effective and powerful screening tool. Standard bulk RNA sequencing (RNA-Seq) enables characterisation of the average expression profiles for individual samples and facilitates identification of the molecular functions associated with genes differentially expressed across conditions. RNA-Seq can also be applied to disentangle splicing variants and discover novel transcripts, thus contributing to a comprehensive understanding of the transcriptome landscape. A closely related technique, single-cell RNA-Seq, has enabled the study of cell-type-specific gene expressions in hundreds to thousands of cells, aiding the exploration of cell heterogeneity. Nowadays, bulk RNA-Seq and single-cell RNA-Seq serve as complementary tools to advance and accelerate the development of transcriptome-based resources. This Collection illustrates how the current global research community makes use of these techniques to address a broad range of questions in life sciences. It demonstrates the usefulness and popularity of high-throughput transcriptomics and presents the best practices and potential issues for the benefit of future end-users.

High-throughput transcriptomics has accelerated our understanding of the variety and complexity of transcriptomes of different tissue types and individual cells. The transcriptome refers to the full range of RNA molecules expressed by a cell, tissue, or organism in a certain physiological condition or at a specific stage of development. The study of the transcriptome is essential to (1) measure the changing levels of expression of each gene under different conditions; (2) characterise transcriptional variants and all possible splicing patterns; (3) identify non-coding RNA (ncRNA) species and study their role in different biological processes. This Collection brings together contributions from scientists addressing diverse biological and medical issues across a broad spectrum of species and cell systems using a variety of transcriptome sequencing techniques.

Since its inception, bulk RNA-sequencing (RNA-Seq) has been the method of choice for studying tissue-level transcriptome changes and has brought a qualitative and quantitative improvement to the transcriptome analysis due to its unlimited dynamic range. Mean expression levels of RNAs isolated from a tissue are detected based on the number of reads mapped to each gene/transcript, and differences in gene expression profiles between samples can then be derived. Hence, the objective of gene/transcript quantification is to identify changes that occur under different experimental conditions, in disease states, and in response to medical treatments, thereby providing a snapshot of the underlying molecular processes taking place.

For example, in a study published as part of the Collection, Austin et al*.* investigated changes in gene expression of human dermal fibroblasts following red light phototherapy and identified several genes as therapeutic targets in the treatment of skin fibrosis^1^. Among these, the metalloproteinase-1 (MMP1) gene, involved in extracellular matrix organization and collagen degradation, was found as one of the critical mediators of fibrotic disease. Showcasing a different application, Sánchez et al*.* clearly showed that transcriptomic changes in the arcuate nucleus and the remaining hypothalamic tissue of heifer calves were associated with enhanced nutrition in early life. Their data can be exploited to design new nutritional regimens capable to promote earlier onset of puberty^2^. RNA-Seq has also been used to examine transcriptional changes in plants; Rathore et al*.* performed a time-series eco-transcriptomics study to monitor temporal changes in the transcriptome of an evergreen shrub in response to changing environmental conditions in natural alpine environments. The authors found that the transcriptome reprogramming of the leaves is greater towards winters and decline during spring and that the de-acclimation period was the transcriptionally most active phase^3^. Finally, RNA-Seq can be successfully combined with computational methods; Yap et al*.* developed a deep learning classifier based on convolutional neural networks that can predict tissue classification from Genotype-Tissue Expression (GTEx) RNA-Seq data, thereby providing more information on tissue-specific gene expression than traditional differential gene expression analysis^4^.

Aside from quantifying transcript expression profiles, random fragmentation used in bulk RNA-Seq library preparation has allowed scientists to detect new features in a transcriptome, such as novel transcripts, RNA isoforms, and gene fusion transcripts. In a paper included in this Collection, Williams et al*.* reported the differential splicing of *CAMK2G* in two mouse models and revealed the relationship between *CAMK2G* and hypoxia inducible factor 1 in the pathogenesis of heart diseases^5^. Estill et al*.* analysed the transcriptome of the nucleus accumbens in the brains of mice treated with cocaine, and found many novel transcripts and splicing sites, revealing information useful in our understanding of addiction and reward^6^.

Although every single cell in an organism carries the same genome content, different cell types can use different portions of the same genome by expressing different subsets of all possible genes. Bulk RNA-Seq does not consider the heterogeneity of a sample and the individual complexity of each cell, so new methods have been refined to enable investigation of the transcriptome at a single-cell level.

Single cell RNA-Seq (scRNA-Seq) has emerged as a breakthrough technology for dissecting tissue heterogeneity, identity, fate, and function by capturing transcriptome profiles from individual cells. Coupled with sophisticated downstream bioinformatic analysis, scRNA-Seq enables investigations of gene expressions in cells. Additionally, scRNA-Seq allows researchers to identify previously unknown cell populations, highlighting the diversity of the molecular processes affecting each individual cell or cell type and revealing differences at the cellular level that are generally obscured by bulk sampling.

Many instruments have been developed with the aim of facilitating the use of scRNA-Seq and the analysis of relevant data. In this Collection, Davey et al*.* described a promising and flexible droplet‐based microfluidics system capable of capturing approximately 50,000 single cells in one run^7^. The authors then used the system to distinguish human cell populations at different phases of the cell cycle and to study transcriptional regulatory networks, thereby pointing out several transcription factors with little or no prior association with distinct cell cycle phases. To address the problems of technical variations between cell subsets introduced by the tissue dissociation procedure, Uniken Venema et al*.* developed a one-step collagenase dissociation protocol suitable for scRNA-Seq and demonstrated its use on cryopreserved gut mucosal biopsies. The protocol showed many advantages, i.e. reduction of times, costs and procedures, prevention of batch effects, high level of reproducibility, and experimental flexibility, over the gold standard two-step collagenase dissociation and three-step protease dissociation protocols^8^.

Most RNA molecules in a transcriptome fall into a broad class known as non-coding RNAs, which have attracted the attention of researchers through their involvement in numerous biological processes. With unprecedented resolution and scale, variations of the RNA-Seq method allow for the identification of a growing number of ncRNAs, and investigation of their potential functions.

Nielsen et al*.* proposed a new method to examine the expression and activity of microRNAs (miRNAs), one of the subtypes of ncRNA, at the single-cell level^9^. As single-cell miRNA sequencing is not yet widespread practice, miRNA activity scores were derived indirectly from the expression profiles of all target mRNAs. The method, based on the enrichment of miRNA binding sites, was then applied to a human and a mouse scRNA-Seq data set, and returned activity measures of several miRNA candidates coherent with literature data. Finally, Su et al*.* studied the positive regulatory role of circular RNAs (circRNAs), another type of ncRNA, in the osteogenic differentiation of mesenchymal stromal cells of the human umbilical cord^10^. The authors identified over 5000 circRNAs, about one percent of which were differentially expressed. Among these, circ‐CTTN was found to play a key role during osteogenic differentiation.

Taken together, this Collection encompasses a broad range of studies using high-throughput transcriptomics alongside other molecular techniques in different research domains, with aims ranging from improving experimental approaches, to developing bioinformatic methods, to applications in real-world scientific questions. This Collection showcases some of the recent developments in transcriptomics studies that we hope will provide guidelines for researchers with an interest in the use of transcriptomics, or other omics techniques, in future studies.

## References

[1] Austin E (2021). Transcriptome analysis of human dermal fibroblasts following red light phototherapy. Sci. Rep..

[2] Sànchez JM (2021). A high plane of nutrition during early life alters the hypothalamic transcriptome of heifer calves. Sci. Rep..

[3] Rathore N (2022). Time-series RNA-Seq transcriptome profiling reveals novel insights about cold acclimation and de-acclimation processes in an evergreen shrub of high altitude. Sci. Rep..

[4] Yap M (2021). Verifying explainability of a deep learning tissue classifier trained on RNA-seq data. Sci. Rep..

[5] Williams AL (2021). Ischemic heart injury leads to HIF1-dependent differential splicing of CaMK2gamma. Sci. Rep..

[6] Estill M (2021). Long read, isoform aware sequencing of mouse nucleus accumbens after chronic cocaine treatment. Sci. Rep..

[7] Davey K (2021). A flexible microfluidic system for single-cell transcriptome profiling elucidates phased transcriptional regulators of cell cycle. Sci. Rep..

[8] Uniken Venema WTC (2022). Gut mucosa dissociation protocols influence cell type proportions and single-cell gene expression levels. Sci. Rep..

[9] Nielsen MM, Pedersen JS (2021). miRNA activity inferred from single cell mRNA expression. Sci. Rep..

[10] Su C, Zheng X, He Y, Long L, Chen W (2021). Transcriptomic profiling and functional prediction reveal aberrant expression of circular RNAs during osteogenic differentiation in human umbilical cord mesenchymal stromal cells. Sci. Rep..

